# Heterogeneity of the Type I Interferon Signature in Rheumatoid Arthritis: A Potential Limitation for Its Use As a Clinical Biomarker

**DOI:** 10.3389/fimmu.2017.02007

**Published:** 2018-01-16

**Authors:** Javier Rodríguez-Carrio, Mercedes Alperi-López, Patricia López, Francisco J. Ballina-García, Ana Suárez

**Affiliations:** ^1^Area of Immunology, Department of Functional Biology, Faculty of Medicine, University of Oviedo, Oviedo, Spain; ^2^Instituto de Investigación Sanitaria del Principado de Asturias (ISPA), Oviedo, Spain; ^3^Department of Rheumatology, Hospital Universitario Central de Asturias, Oviedo, Spain

**Keywords:** arthritis, interferon, IFN signature, biomarker, autoimmunity

## Abstract

**Introduction:**

An increased expression of interferon (IFN)-responding genes (IRGs), the so-called IFN signature, has been reported in rheumatoid arthritis (RA). However, some controversy exists concerning its clinical relevance. The main aim of this study is to evaluate whether quantitative and qualitative differences in the activation of the IFN pathway may account for these findings.

**Methods:**

The expression of IFN-induced protein 44 (IFI44), IFN-induced protein 44 like (IFI44L), IFN alpha inducible protein 6, and MX dynamin-like GTPase 1 (MX1) was determined in peripheral blood in 98 RA patients (IFI6) and 28 controls. RA patients were classified into groups according to their clinical stage and treatments received: very early RA (VERA), biological disease-modifying antirheumatic drug (bDMARD) naive, and bDMARD. An additional group of 13 RA patients candidates for tumor necrosis factor alpha (TNFα) blockade was also recruited. The associations among IRGs were evaluated by network and principal component analyses.

**Results:**

The expression of all IRGs was increased in RA to different levels. The IFN score was increased in all RA groups (VERA, bDMARD-naïve, and bDMARD), but important differences in their degree of activation and in the relationships among IRGs were observed. The IFN score correlated with the accumulated disease activity score 28-joints, and it was found to be a predictor of clinical outcome in VERA. No differences in the IFN score were observed between the bDMARD-naive and bDMARD groups, but opposite associations with the clinical parameters were noted. Interestingly, the correlations among IRGs delineate different pictures between these two groups. The IFN score at baseline predicted poor clinical outcome upon TNFα blockade. Although no absolute changes in the IFN score were found, TNFα-blockade shifted the associations among IRGs.

**Conclusion:**

A certain heterogeneity within the IFN signature can be recognized in RA, depending on the clinical stage. The structure of the IFN signature may be a potential explanation for the controversy in this field and must be considered to decipher its clinical relevancein RA.

## Introduction

Rheumatoid arthritis (RA) is a chronic systemic autoimmune condition hallmarked by joint inflammation and destruction. A number of immune mediators have been linked to RA pathogenesis, including adaptive and innate components. A growing body of evidence supports an emerging role for type I interferons (IFNs) (1). Due to their immunomodulatory effects (2), the type I IFNs are thought to prompt the breakdown of tolerance and the subsequent perpetuation of autoimmune phenomena (3, 4). Actually, several IFN-related genes have been identified as risk loci for RA (3), and development of arthritis after treatment with IFNα has been extensively documented (5, 6).

Signaling through the type I IFN pathway leads to an increased expression of several IFN-responding genes (IRGs). This global expression profile, referred to as the “IFN signature,” has been found in peripheral blood in a subset of RA patients [from 25 to 65% (7–10)]. Moreover, increased serum levels of IFNα have been demonstrated in RA (11). Elevated IFNα serum levels in the synovial fluid and IFN signature in the synovial membrane have also been reported (8, 12).

Although the potential role of the type I IFN as biomarkers has been investigated with enormous interest, the findings reported until date are contradictory and the current evidence is limited. On the one hand, inconclusive results of the association between the IFN score and clinical features have been reported (9, 10, 13, 14). On the other hand, longitudinal changes of the IFN score have been described, partly attributed to the use of different immunomodulatory drugs (15–17). In addition, the majority of the studies were focused on patients with established disease, whereas a major knowledge gap exists for the role of the IFN signature in (very) early RA. Finally, a physiological diversification of the type I IFN response in different autoimmune diseases has been described, hence suggesting that different pathogenic roles for the type I IFNs may be expected in different clinical contexts (18). However, whether this can be applied to a single disease remains unknown.

Therefore, it is tempting to speculate that not only the degree of activation but also the composition of the IFN response could be relevant for its role as a biomarker. Taken all these ideas into account, we hypothesized that certain heterogeneity within the type I IFN signature in RA may impair its applicability as a biomarker, hence explaining the controversy reported in previous works. Thus, in this study, we aimed to analyze the potential associations between the IFN score and clinical features in RA patients depending on their clinical stage [from very early RA (VERA) to established disease], with a focus on the relationships among IRGs.

## Materials and Methods

### Ethical Approval

The study was approved by the Institutional Review Board (Comité de Ética de Investigación Clínica del Principado de Asturias) in compliance with the Declaration of Helsinki. All study subjects gave written informed consent.

### Patients and Controls

Our study involved 98 RA patients [2010 ACR/European league against rheumatism (EULAR) classification criteria] recruited from the Department of Rheumatology at Hospital Universitario Central de Asturias. A complete clinical examination, including disease activity score 28-joints (DAS28) and health assessment questionnaire (HAQ) calculations, was performed on all patients during the clinical appointment. Patients recruited at onset and not being previously exposed to any treatment were considered as VERA. These patients were prospectively followed up for 1 year, and clinical outcomes were registered at 6 and 12 months. DAS28 score accumulated over 1 year was calculated (19). Clinical management was performed according to EULAR recommendations (20). In addition, a group of 13 biologicals-naive RA patients [12 women; median age, 43 (range, 30–65), DAS28 5.08 (1.93), 38.5% RF+, 46.1% ACPA+], candidates for tumor necrosis factor alpha (TNFα)-blockers was prospectively followed up for 3 months. A blood sample was obtained before and 3 months after the initiation of the TNFα blockade therapy. The clinical response was evaluated by EULAR criteria (21). Patients exhibiting a good response were compared to those with moderate or no response.

Simultaneously, 28 gender- and age-matched healthy controls (HCs) were recruited from the same population. A blood sample was collected from all individuals by venipuncture.

### RNA Isolation and RT-PCR

Blood samples were immediately processed after extraction. Whole blood was stabilized with RNA Stabilization Reagent for Blood/Bone Marrow (Roche, Germany) according to the manufacturer’s instructions and stored at −20°C. Then samples were thawed at room temperature, and mRNA was isolated using the mRNA Isolation Kit for Blood/Bone Marrow (Roche), according to the protocol provided by the manufacturer. Reverse transcription was carried out using Transcription First Strand cDNA Synthesis Kit (Roche).

### Real-time PCR

Gene expression was evaluated with TaqMan pre-designed assays for the following genes: IFN-induced protein 44 (IFI44; ref. Hs00197427_m1), IFN-induced protein 44 like (IFI44L; ref. Hs00915292_m1), MX dynamin-like GTPase 1 (MX1; ref. Hs00895608_m1), and IFN alpha inducible protein 6 (IFI6; ref. Hs00242571_m1). Reactions were performed in TaqMan^®^ Gene Expression Master Mix. Real-time quantitative PCR was performed in an ABI Prism HT7900 (Applied Biosystems, Germany) instrument, and Ct values were analyzed with the software SDS 2.3. All samples were assayed by triplicate, and the average was used. Expression levels were evaluated by the 2^−ΔCt^ method, using the GAPDH gene expression as the housekeeping to normalize Ct values. The expression levels were log transformed.

### Statistical Analyses

Continuous variables were summarized as median (interquartile range) or mean ± SD, whereas *n* (%) was used for categorical ones. Differences between groups were analyzed by Mann–Whitney *U*-test, Kruskal–Wallis (with Dunn–Bonferroni correction for multiple comparisons), or χ^2^ tests, as appropriate. Wilcoxon test was used for paired samples. The size effect of the differences was evaluated by Hedges’g statistic (22). Correlations were assessed by Spearman ranks test. The associations of continuous variables adjusted for confounders were analyzed by multiple regression models, and B coefficients (B) with 95% confidence intervals (CIs) were calculated. The discriminative capacity was studied using receiving operator characteristics analyses, and the area under the curve (AUC) was computed. *Z*-scores were calculated for each IRG from the distribution found in the whole population. Principal component analyses (correlation method) were performed with the individual IRGs, and biplots were generated to visualize the associations among IRG in the different groups of patients. Since strong correlations among IRGs were observed, an IFN score was calculated by averaging all IRGs per sample. *P* < 0.050 was considered as statistically significant. Statistical analyses were performed using SPSS 22.0, R 3.3.1 and GraphPad Prism 5.0 for Windows.

## Results

### IRG Genes and IFN Score: Quantitative and Qualitative Approaches

**Figure 1:**
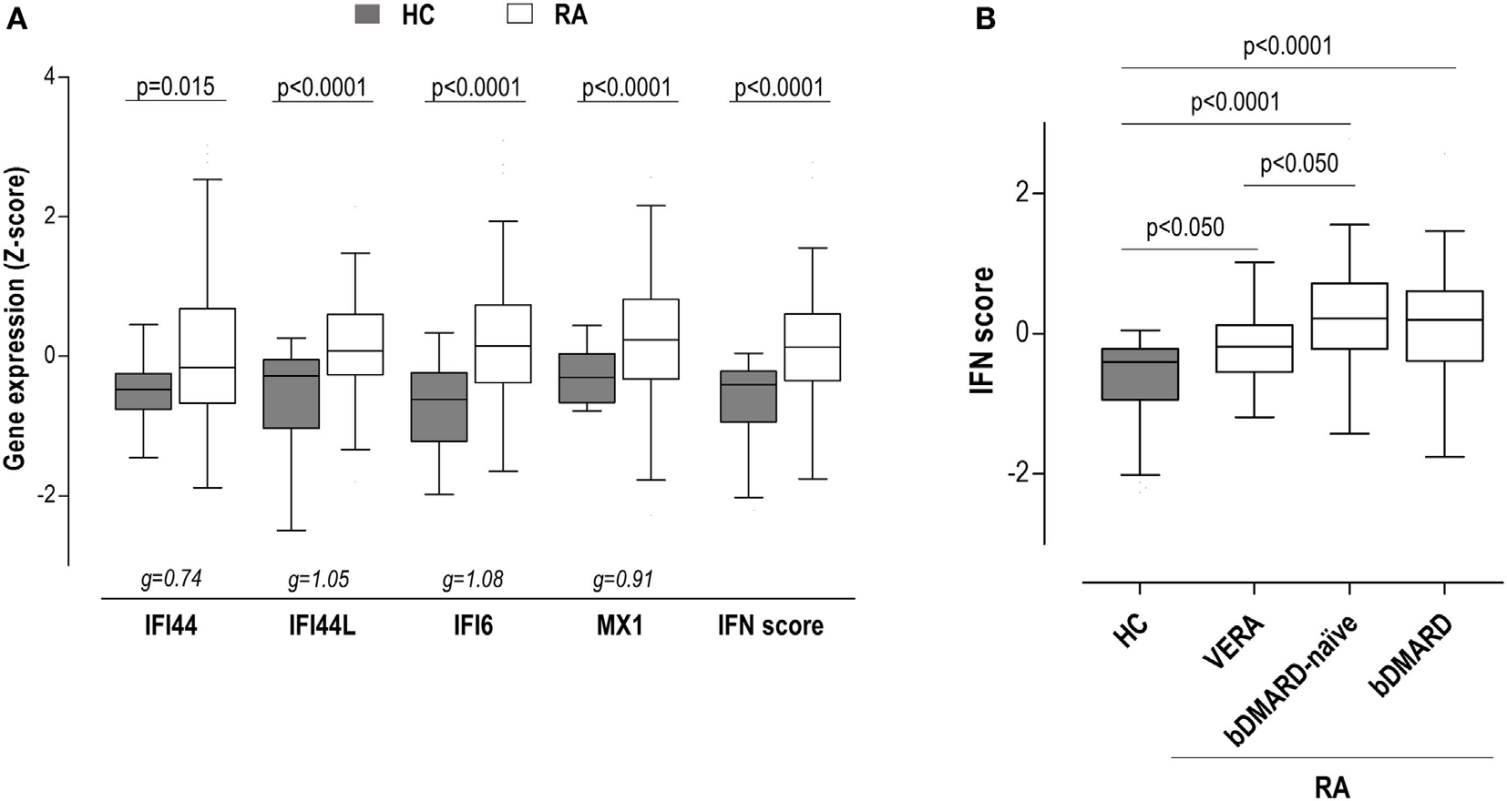
Interferon (IFN)-responding gene (IRG) expression and IFN score in rheumatoid arthritis (RA) patients. **(A)** The expression of the individual IRGs [IFN-induced protein 44 (IFI44), IFN-induced protein 44 like (IFI44L), IFN alpha inducible protein 6 (IFI6), and MX dynamin-like GTPase 1 (MX1)] and the levels of the IFN score were compared between healthy control (HC) (gray boxes) and RA patients (white boxes). **(B)** The levels of the IFN score were compared between HC and RA patients according to the different clinical stages. Boxes represent 25th and 75th percentiles, whereas whiskers represent minimum and maximum values. Statistical analyses were performed by Kruskal–Wallis with Dunn–Bonferroni tests for multiple comparisons. *P* values correspond to those obtained in the multiple comparisons tests. For the analysis of individual IRGs, Hedges’g statistics (at the bottom of the graph) was included to account for the differential size effects observed across IRGs.

To gain insight into the type I IFN signature in RA, the expression of IRGs, either independently or as a whole in a composite IFN score, was quantified in 98 RA patients and 28 HC (Table 1). All IRGs were upregulated in RA (Figure 1A), although a less-pronounced difference was observed in IFI44. A composite IFN score was computed as previously described, and a higher value was found in RA (Figure 1A).

**Table 1:** Characteristics of the subjects recruited in this study.

	HC (*n* = 28)	RA (*n* = 98)	*P* value
**Demographical features**			
Age (years); median (range)	49.38 (35.17–60.17)	52.93 (22.00–65.10)	0.174
Gender (f/m)	20/8	79/19	0.334
**Disease features**			
Disease duration (years)		4.00 (7.08)	
Age at diagnosis (years); median (range)		47.37 (19.00–65.00)	
Disease activity (DAS28)		4.01 (2.10)	
Tender joint count		3.00 (7.00)	
Swollen joint count		2.00 (5.00)	
Patient global assessment (0–100)		50.00 (35.00)	
Erythrocyte sedimentation rate (mm/h)		18.00 (29.00)	
C-reactive protein (mg/l)		2.30 (4.50)	
Health assessment questionnaire (0–3)		1.00 (1.16)	
RF (+), *n* (%)		58 (59.1)	
ACPA (+), *n* (%)		61 (62.2)	
Erosive disease, *n* (%)		41 (41.8)	
**Treatments, *n* (%)**			
None		18 (18.3)	
Glucocorticoids		56 (57.1)	
Methotrexate		65 (66.3)	
Tumor necrosis factor alpha blockers		36 (36.7)	

*Variables were summarized as median (interquartile range) or n (%), as appropriate, unless otherwise stated. Differences in demographic parameters were assessed by Mann–Whitney U-tests or χ^2^ tests, according to the distribution of the variables*.

Importantly, certain heterogeneity among RA patients was observed. Therefore, we aimed to analyze whether the IFN score may differ according to the clinical stage of RA. Then patients were classified into three groups: VERA (patients recruited at onset, not being exposed to any treatment), biological disease-modifying antirheumatic drug (bDMARD)-naive [patients on conventional synthetic DMARDs (csDMARD) treatment alone or in combination—glucocorticoids and/or methotrexate—not being previously exposed to any bDMARD], and bDMARD (patients on bDMARD therapy—all under TNFi treatment—with previous no response to csDMARD) (Table S1 in Supplementary Material). Although the IFN score was increased in all groups, quantitative differences were observed between VERA patients and their established counterparts (Figure 1B), hence indicating that the level of IRGs expression differed according to the disease course. No differences were found with the use of bDMARDs. Leukocyte populations did not exhibit notable differences among the groups analyzed (Table S2 in Supplementary Material). Only a slight increase in neutrophil count was observed in VERA patients. To exclude a potential confounding effect of the leukocyte composition, the IFN score was corrected by the neutrophil-to-lymphocyte ratio, and the differences among groups remained unchanged. Similarly, the IFN score did not correlate with any of the leukocyte populations nor in the whole RA population neither in the different clinical stages. Overall, a major effect of leukocyte composition on the IFN score can be ruled out.

**Figure 2:**
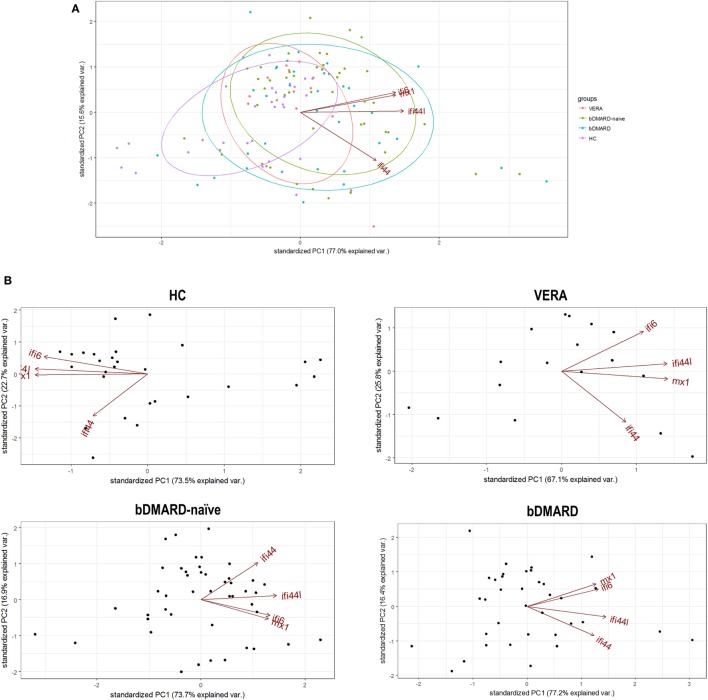
Analysis of the associations among interferon (IFN)-responding genes (IRGs) by PCA. **(A)** Biplot from the PCA (correlation method) conducted on all the groups analyzed [healthy control (HC), purple; VERA, red; biological disease-modifying antirheumatic drug (bDMARD)-naive, green; and bDMARD, blue]. Arrows represent the original variables. The angles between the arrows represent their correlation. Ellipses are drawn for each group (probability set as 0.68, by default). KMO = 0.705, Bartlett sphericity test *P* = 4 × 10^−8^, determinant = 0.032. **(B)** PCA conducted individually for each group of individuals included in the study: HC [KMO = 0.695, Bartlett sphericity test *P* = 1.5 × 10^−25^, determinant = 0.012], VERA [KMO = 0.734, Bartlett sphericity *P* = 2 × 10^−7^, determinant = 0.051], bDMARD-naive [KMO = 0.700, Bartlett sphericity test *P* = 1 × 10^−23^, determinant = 0.053], and bDMARD [KMO = 0.642, Bartlett sphericity test *P* = 2 × 10^−25^, determinant = 0.020].

Apart from quantitative differences, we aimed to evaluate whether qualitative differences, that is, distinct associations among IRGs, can be also found. To this aim, a PCA approach was conducted, and biplots were produced to visualize the potential associations among IRGs. First, a global PCA including all RA patients and HC was performed. Although a significant overlap was detected, differences were noted among groups (Figure 2A). The biggest differences were observed between HC and RA patients. The ellipse from VERA patients lie between those of HC and established RA groups. Again, IFI44 showed an outlier position in the graph compared to the rest of IRGs. However, certain dispersion was noted. It is important to note that differences observed in the IRGs expression and the distinct sample sizes could also limit a proper appraisal of the qualitative differences among groups. Therefore, additional analyses to gain insight into these differences were warranted.

**Figure 3:**
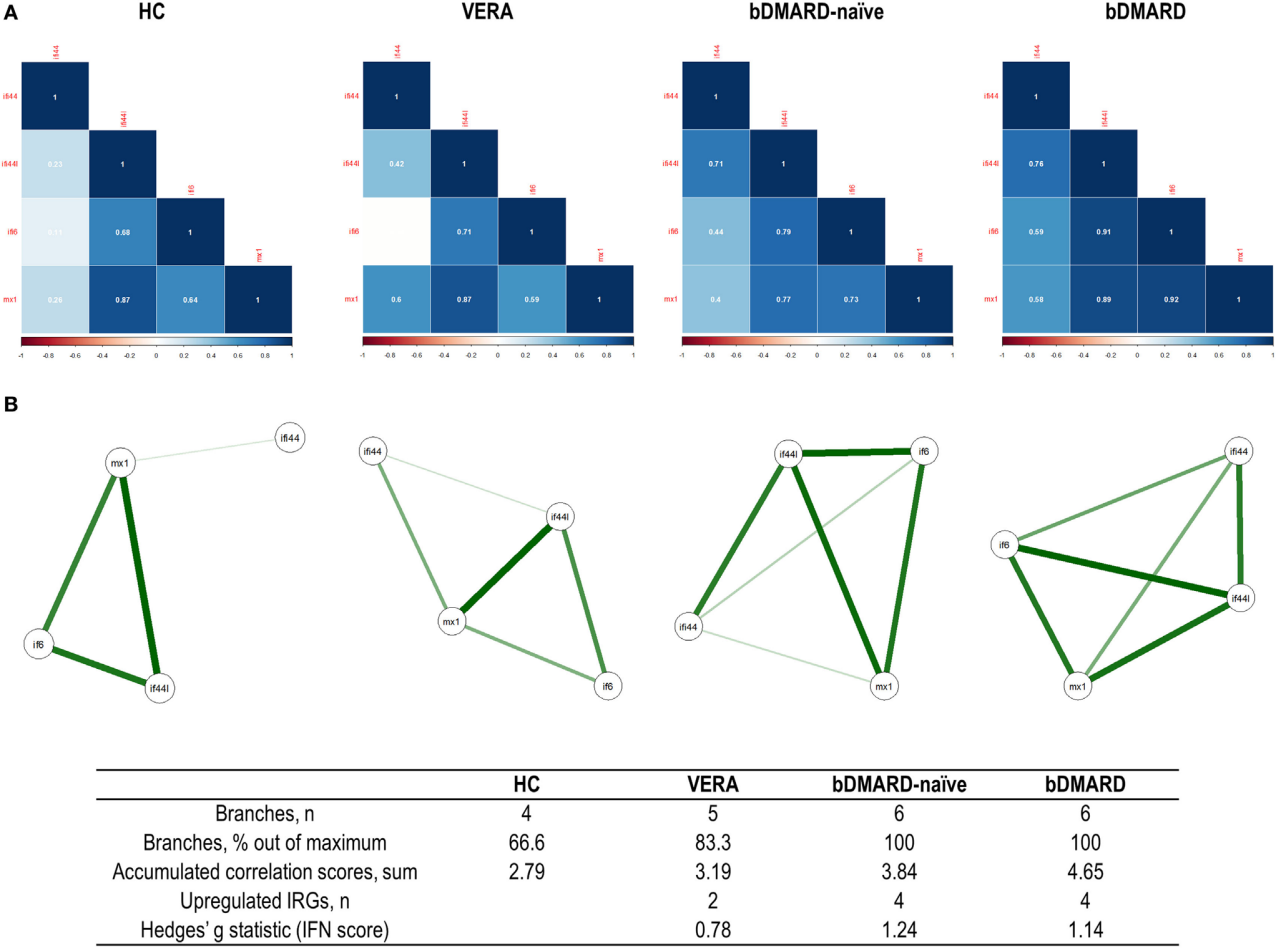
Analysis of the correlations and structure of the interferon (IFN)-responding genes (IRGs) networks. **(A)** Analyses of the correlations among IRG in the different groups studied. Correlation matrices were plotted in 2 × 2 correlograms. Correlation coefficients were depicted in white. Color of the tiles is proportional to the strength of the correlation (color scale is represented at the bottom of each grap). **(B)** Network analyses of the IRGs in the groups studied. Each node corresponds to an IRG, and the lines illustrate the strength (width) and sign (green, positive; red, negative) of the correlations between each pair of variables. The interferon (IFN) network in healthy control (HC) is characterized by a group of three IRGs closely correlated [IFN-induced protein 44 like (IFI44L), MX dynamin-like GTPase 1, and IFN alpha inducible protein 6 (IFI6)], almost not related to IFI44L. Stronger correlations were observed in very early rheumatoid arthritis (VERA) and rheumatoid arthritis (RA) established groups compared to HC, and a more complex, intricate network was observed in biological disease-modifying antirheumatic drug (bDMARD) patients, with a closer location of all IRGs and a strong correlation between IFN-induced protein 44 (IFI44) and IFI44L. Similarly, a correlation between IFI6 and IFI44 is differentially expressed in established RA, compared to VERA or HC. Additional information on the network structure and its overall activation was summarized in the table at the bottom of the figure.

Then independent biplots were generated for each group (Figure 2B). Based on the angles between the vectors of the IRGs, these analyses revealed that distinct pictures hallmarked the clinical stages analyzed. First, remarkable differences were observed between HC and RA patients. Next, a distinct picture was found in the VERA group compared to both the bDMARD-naive and bDMARD groups, depending on the relative position of the IRGs. A shortening of the angles between IFI6 and MX1 as well as between IFI44 and IFI44L was seen in the established groups, especially in the bDMARD group. Furthermore, the associations among IRGs were plotted in correlation graphs (Figure 3A). This approach revealed that the associations among IRG were not homogeneous in the groups analyzed and, more importantly, IRGs exhibited a higher overall degree of correlation in patients with established RA, especially in the bDMARD group. Finally, network graphs were generated to visualize the interactions among independent genes (Figure 3B). Notably, the structure of the network differed among the groups analyzed, IRGs describing different grouping patterns. Networks seem to show a progressive change from HC, where a weaker network (that is, with subtle links among IRGs) is observed, toward a strengthening of these links along the clinical stages, with an enhanced overall degree of correlation being found in bDMARD patients, hence confirming our previous observations.

Overall, these results confirm quantitative and qualitative changes in the activation of the type I IFN pathway in RA. Differential profiles of correlations among IRGs can be observed according to disease status, hence pointing to certain heterogeneity within the IFN signature.

### IFN Signature As Predictor of Clinical Response in VERA

Next, we studied the clinical relevance of the IFN signature in the distinct groups. First, the potential role of the IFN signature as predictor of the clinical outcome in untreated, VERA patients was assessed. Therefore, patients were prospectively followed up for 1 year and disease activity and response to csDMARD treatment (glucocorticoids and methotrexate in combination) were registered at 6 (T6) and 12 months (T12).

**Figure 4:**
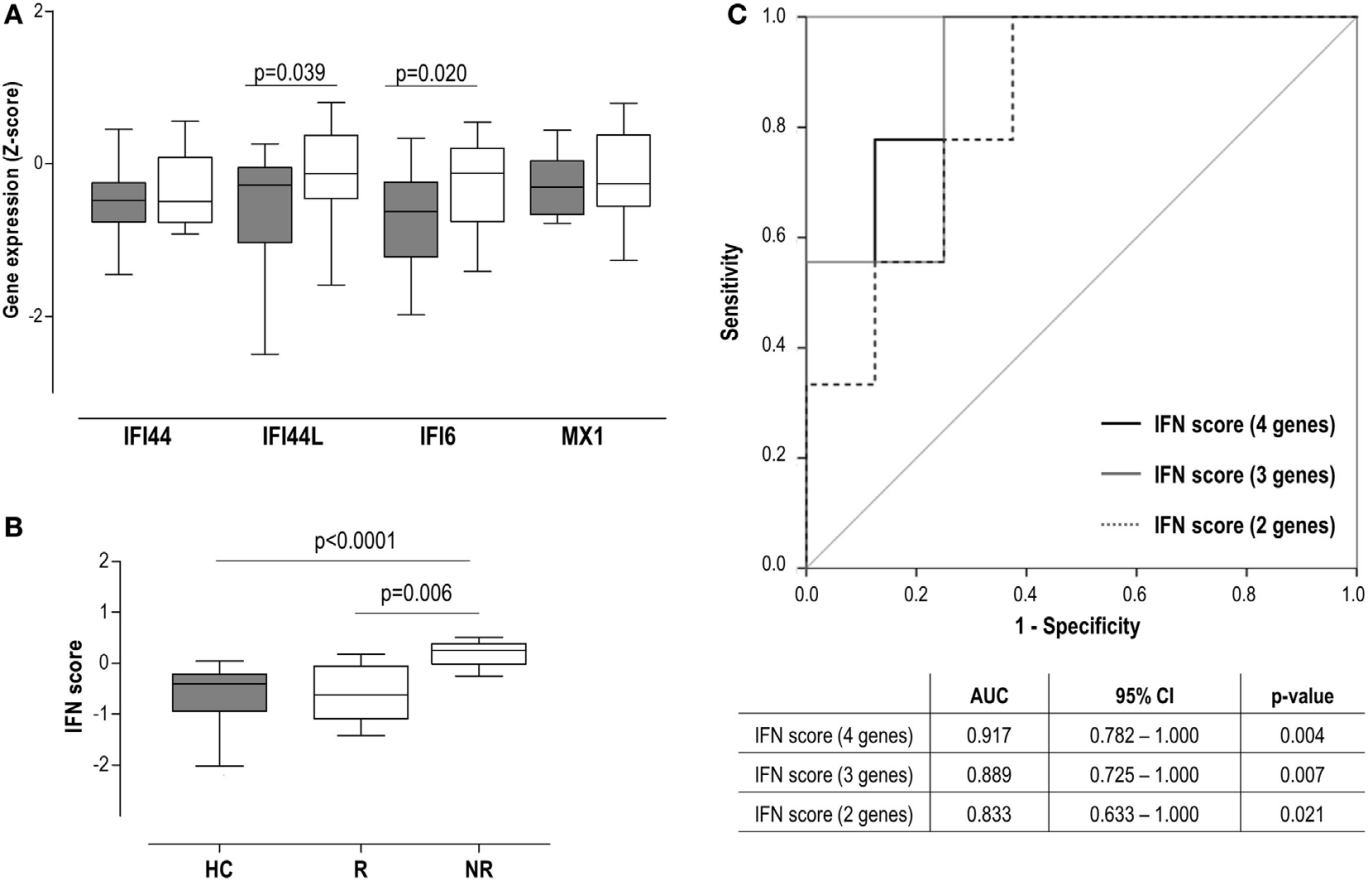
Interferon (IFN) signature as a biomarker of clinical outcome in very early rheumatoid arthritis (VERA) patients. **(A)** Analysis of the individual IFN-responding genes expression in VERA patients (white boxes) compared to healthy control (HC) (gray boxes). **(B)** Differences in the IFN score among HC and rheumatoid arthritis patients classified as responders (R) or non-responders (NRs) according to European league against rheumatism criteria after 6 months. Boxes represent 25th and 75th percentiles, whereas whiskers represent minimum and maximum values. Statistical analyses were performed by Kruskal–Wallis with Dunn–Bonferroni tests for multiple comparisons. *P* values correspond to those obtained in the multiple comparisons tests. **(C)** Area under the curve (AUC) receiving operator characteristic analysis of the IFN score to predict response at 12 months. AUCs, 95% confidence intervals (CIs), and *P* values are shown in the table for the IFN score: four genes [IFN-induced protein 44 (IFI44), IFN-induced protein 44 like (IFI44L), IFN alpha inducible protein 6 (IFI6), and MX dynamin-like GTPase 1 (MX1)], three genes (IFI44L, IFI6, and MX1), and two genes (IFI44L and IFI6).

Although the IFN score at baseline (BL) was observed to be increased already in this very early stage (Figure 1B), when IRGs were analyzed, only differences in IFI44L and IFI6 were observed (Figure 4A). Regarding clinical features, a positive correlation between IFN score and anticitrullinated peptide antibody (ACPA) titer was found (*r* = 0.532, *P* = 0.034).

Interestingly, our analyses did not retrieve an association between the IFN score and the DAS28 at sampling (*r* = −0.055, *P* = 0.835), but with DAS28 at T6 (*r* = 0.620, *P* = 0.008) and T12 (*r* = 0.552, *P* = 0.041). Consequently, the IFN score at BL was positively associated with the accumulated DAS28 over 1 year (AUC DAS28: *r* = 0.593, *P* = 0.025). A multivariate analysis including IFN score, ACPA, rheumatoid factor (RF), and gender revealed that IFN score at BL may be the only independent predictor of accumulated DAS28 [B (95% CI): 12.883 (−0.381 to 26.147), *P* = 0.051], although statistical significance was not reached. Then patients were grouped according to their clinical response at T6 [responders (R), *n* = 8] and T12 (R, *n* = 10). A higher IFN score at diagnosis was observed in patients who exhibited a poor clinical response at T6 (Figure 4B), compared to either good responders or HC. Equivalent results were observed at 12 months [non-responder (NR) vs R: 0.17 (0.82) vs −0.44 (0.77), *P* = 0.039; vs HC: −0.40 (72), *P* = 0.003]. This association with the clinical outcome was observed when IRGs were analyzed individually except for IFI44 (Figure S1 in Supplementary Material), both at T6 and T12. Interestingly, even in the case of MX1, despite not being increased compared to HC, increased levels were observed in NR. Next, the ability of the IFN score to discriminate between responders and non-responders was evaluated by COR curves. Accordingly, IFN score showed a good discriminative capacity. Exclusion of the IFI44 expression [IFN score (3 genes)] from the composite score did not substantially change the results. Similarly, a lower but still good discriminative capacity was found when only IFI6 and IFI44L were retained in the IFN score (Figure 4C).

In sum, a high IFN score at diagnosis in untreated RA patients is linked to a poor clinical outcome, thus shedding some light into their potential clinical implications as a biomarker.

### IFN Signature in Established RA

Next, an analysis of the potential associations between the IFN score and clinical features in patients with established RA was performed.

The IFN score was not correlated with DAS28, disease duration, or RF/ACPA positivity in the bDMARD-naive group. In addition, no associations with GC or MTX treatment were found. The clinical response of these patients to csDMARD therapy was monitored during 1 year, and five patients were switched to a bDMARD treatment due to clinical inefficacy of csDMARD treatment. The analysis of these patients revealed that IFN score at study entry did not differ compared to those who continued on csDMARDs [−0.13 (0.89) vs 0.27 (0.89), *P* = 0.140].

Then the associations between the IFN score and clinical features were analyzed in patients undergoing bDMARD treatment (all anti-TNFα agents). Surprisingly, a negative correlation between IFN score and DAS28 was observed (*r* = −0.358, *P* = 0.032), but no associations were found with disease duration or HAQ. When individual genes were analyzed, MX1 showed a stronger correlation with DAS28 (*r* = −0.459, *P* = 0.005) compared to the other IRGs (IFI44: *r* = −0.104, *P* = 0.546; IFI44L: *r* = −0.384, *P* = 0.021 and IFI6: *r* = −0.394, *P* = 0.017). In addition, negative associations with RF titer (*r* = −0.565, *P* < 0.001) and trend with ACPA levels (*r* = −0.302, *P* = 0.087) were found for the IFN score. Importantly, no effect of GC or MTX co-treatment was observed.

As a conclusion, the IFN score was not associated with the clinical response to csDMARD in established RA. Surprisingly, opposed associations with clinical features were noted between bDMARD naive and bDMARD patients, a negative correlation with disease activity being found in the latter. Whether bDMARD-induced qualitative changes on the IFN signature underlie these contrary results remains to be elucidated.

### Changes in the IFN Score upon TNFα Blockade

Although a similar IFN score between bDMARD-naive and their bDMARD-treated counterparts was found, its clinical relevance was notably different between them. Then we hypothesized that qualitative changes within the IFN score composition occurring upon TNFα blockade may underlie these findings. To further explore this idea, the IFN score was prospectively analyzed in a group of 13 biological-naive RA patients at BL and after 3 months upon TNFα blockade [posttreatment (PT)].

**Figure 5:**
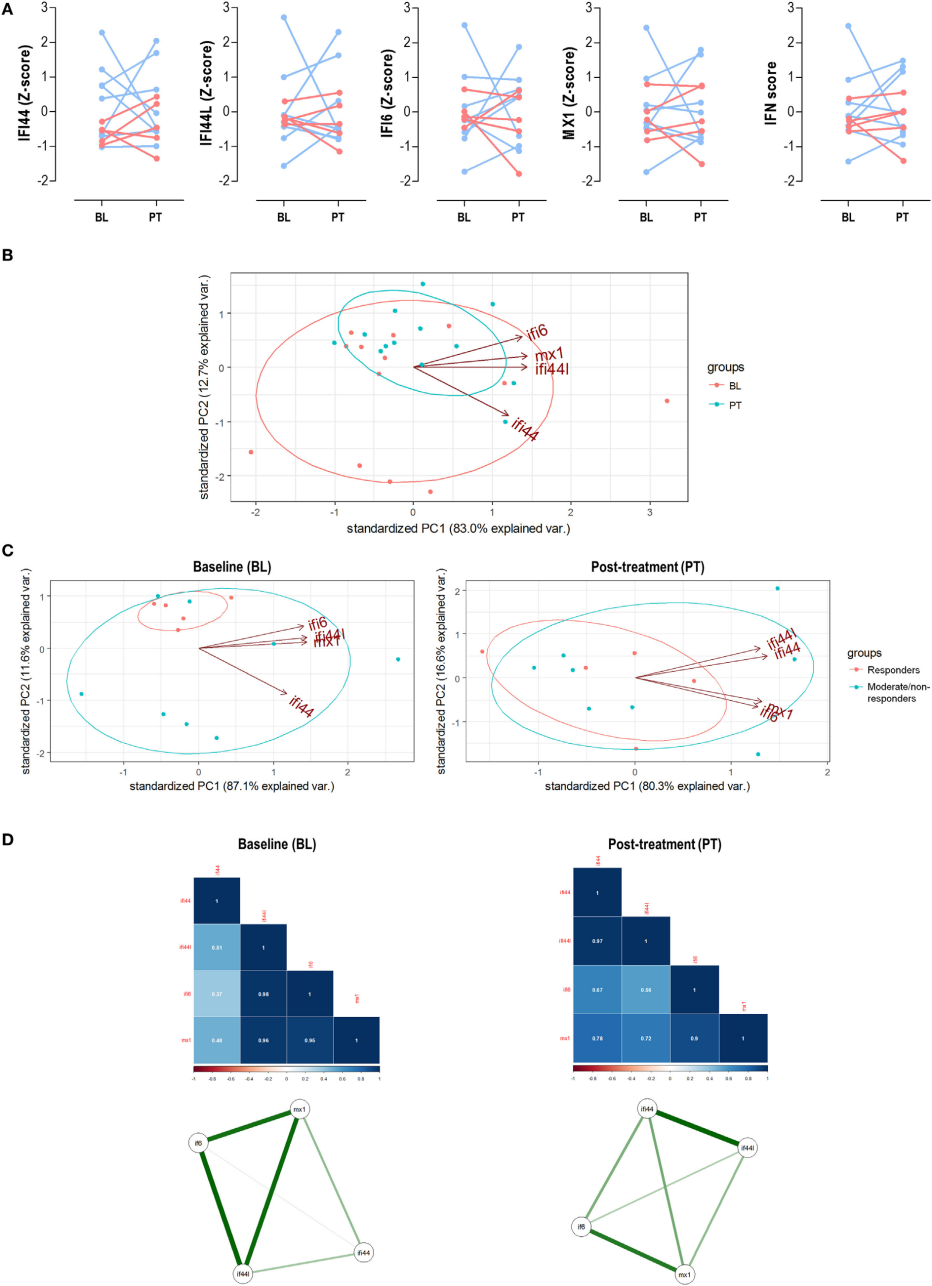
Analysis of the interferon (IFN) signature upon tumor necrosis factor alpha (TNFα) blockade. **(A)** Paired analyses of the expression of the individual IFN-responding genes (IRGs) and IFN score at baseline (BL) and posttreatment (PT) with anti-tumor necrosis factor alpha (TNFα) in 13 patients prospectively followed up. Patients were represented in red (responders) and blue (moderate/non-responders). Statistical analyses were performed by Wilcoxon test. **(B)** Biplot from the PCA (correlation method) conducted on the—BL, red; PT, blue—samples from rheumatoid arthritis patients. Ellipses are drawn for each group (probability set as 0.68, by default). **(C)** PCA independently conducted for BL and PT samples to evaluate changes in the IRG occurring upon TNFα blockade. **(D)** Correlation matrices and network analyses of the IRGs in the BL and PT samples. Stronger associations among IFN-induced protein 44 like (IFI44L), IFN alpha inducible protein 6 (IFI6), and MX dynamin-like GTPase 1 (MX1) were found in the BL samples, with a farther location for IFN-induced protein 44 (IFI44). However, a more uniform pattern among IRGs was found after TNFα blockade (PT), pointing toward two IRG clusters (IFI44 + IFI44L and IFI6 + MX1). These results confirmed those obtained in the PCA and are in line with the findings from the cross-sectional analysis (Figure 2).

On the one hand, neither the IFN score nor individual IRGs changed upon TNFα blockade (Figure 5A). No changes in leukocytes, neutrophils, lymphocytes, or monocytes were observed (all *P* > 0.050). No association between IFN score and DAS28 at sampling was found in none of the time points analyzed. However, IFN score at BL was an independent predictor of DAS28 after treatment [B (95% CI): 0.577 (0.052–1.102), *P* = 0.035] after adjusting for gender, RF, and ACPA positivity. However, when patients were grouped according to their clinical outcome, no differences in the IRGs expression levels were observed (Figure 4A) (Table S3 in Supplementary Material).

Next, we evaluated the associations among IRGs upon TNFα blockade. First, a PCA conducted with IRGs from RA patients before and after TNFα blockade (Figure 5B) revealed that independent groups could not be identified, which is in line with the lack of absolute differences observed. Actually a notable overlap was found, similar to that of bDMARD-naive and bDMARD groups in Figure 2A. Nevertheless, BL and PT showed different distributions, which may be attributed to distinct genes hallmarking each group. In fact, when the associations among individual IRGs were compared between BL and PT samples, different IRGs profiles were detected (Figure 5C). The correlation graphs and the network analyses (Figure 5D) supported changes in the correlation profiles among the IRGs and confirmed different structural organization of the IFN signature before and after TNFα blockade. Interestingly, these observations also paralleled those obtained in our cross-sectional analysis (Figure 3).

Taken together, these results confirm that TNFα blockade lead to profound qualitative changes within the coordinate expression of IRGs rather than absolute changes in the gene expression levels. These qualitative changes may underlie the different associations observed with clinical features.

## Discussion

Although a compelling body of evidence highlights a potential role for type I IFNs in RA, its clinical relevance remains poorly understood. This study sheds new light on the type I IFN activation in RA. Our findings revealed that the IFN signature is present already in the very early stage of the disease, and quantitative and qualitative changes occur along the disease course. Although the type I IFN score at onset can be proposed as a biomarker of clinical outcome, a different picture is observed in patients with established disease, especially in patients undergoing bDMARD treatment. Distinct associations among IRGs paralleled these observations. Overall, our results add some complexity to this field and suggest that the IFN signature(s) are less uniform and simple than currently considered.

An important finding from our study is the characterization of the type I IFN signature during the earliest phase of RA. Although several studies have focused on the type I IFN signature in patients with established disease (9, 10, 13, 14), this is the first study where IRGs are quantitatively and qualitatively analyzed in a group of untreated, VERA patients, and its clinical relevance is prospectively assessed. A potential role for type I IFNs during the early stage of the disease can be expected. These findings are in line with previous evidence linking arthritis development to the use of IFNα as a therapeutic agent (5, 6). In addition, it has been reported that activation of the type I IFN program in arthralgia patients is associated with the progression of arthritis (23, 24). In this sense, our results not only found an activation of the type I IFN signature but also go further by pointing to a clinical relevance for the IFN score at disease onset as a biomarker of clinical response. Equivalent results were recently reported by Cooles et al. in relation to the clinical response to initial therapy with csDMARDs (25). However, patients undergoing glucocorticoid treatment were excluded from the analysis to avoid a potential confounding effect. Then, our findings expand the previous evidence, since glucocorticoid treatment did not interfere with the prognostic capacity of the IFN score in VERA patients. Further studies are warranted to elucidate the clinical significance of the IFN signature in the long term.

Another remarkable finding from our study is the complexity observed within the IFN signature along the disease course. The associations among individual IRGs were not homogeneous in RA, but differed according to the disease course. Then, it seems that the type I IFN signature is not the mere result of a global overactivation, but specific expression programs may be detected. The identification of the main genes hallmarking the IFN signature and, more importantly, the distinct associations found among them will be a key to understand the relevance of the IFN signature. Although most of the previous studies have focused on the first point, less attention has been paid to the latter. Interestingly, it has been reported that the overall state of correlation and co-regulation phenomena are crucial to the type I IFN signature (26). Similarly, Somers et al. found prominent differences among IRGs in lupus when analyzed on the basis of a PCA, with three clusters being defined by five IRGs (27). However, whether a similar picture could be found in RA remained unknown. Our approach led us to propose that the IFN signature exhibited quantitative and qualitative changes among the different clinical stages in RA. Different profiles within the IFN signature were observed, suggesting that distinct IRGs (or IRGs clusters) were responsible for the IFN signature in each clinical stage. This picture may be related to the existence of co-regulation and co-expression mechanisms within gene expression profiles. In this sense, a proper analysis of the associations among genes can help to delineate functional biological programs with clinical relevance (26, 28), such as the response to a therapy or the disease aggravation, hence supporting the need of capturing such heterogeneity.

Therefore, it seems plausible that heterogeneity of the IFN signature may impair its clinical applicability. This notion was proposed in a recent article, where the IFN signature was observed to be affected by some csDMARDs, with the exception of MTX (14). This is in line, at least in part, with the results herein reported. However, our results add to the current knowledge by studying the associations among IRGs. Overall, these findings may explain why the type I IFN signature was associated with clinical features in very early, untreated RA patients, but not in those on csDMARD treatment. Similar conclusions have been recently published by other authors (25). Overall, it is feasible that the distinct profiles of the IFN response observed may be a source of controversy in relation to previous studies. Although some confounders, which can alter the IFN response have been reported (14, 25, 29, 30), they were restricted to the degree of activation. However, this is the first time that a qualitative insight is addressed.

An equivalent scenario is depicted in the comparison between biological-naive patients and their biological-treated counterparts. Despite not differing in their absolute IFN score, divergent associations with clinical parameters were registered in the cross-sectional analysis. Similar results were observed in the prospective subgroup. Interestingly, a poor outcome upon TNFα blockade had been linked to the activation of the IFN pathway in RA (15), although no differences were observed upon treatment, which is in line with our findings. In that article, a large interindividual variation was observed (15), being attributed to the result of different regulatory mechanisms. However, this heterogeneity was not approached. On the other hand, distinct profiles among IRG were found before and after the exposure to this therapy. Importantly, the IFN score was negatively associated with different clinical features in bDMARD-treated patients, thus suggesting a potential regulatory or suppressive impact of the IFN score in these patients. Among the individual genes analyzed, stronger associations were observed for MX1, hence pointing to this gene as a potential driver of this effect. Interestingly, MX1 is known to be a gene target gene of IFNβ (31, 32). Consequently, it may be conceivable that bDMARD treatment could be related to an IFNβ-related, rather than a IFNα-related signature. Actually, both IFNs have been described to contribute to the IFN signature in RA (33, 34) and the IFNβ/α ratio has been reported to be a predictor of good therapeutic response in anti-TNF-treated patients (33). Recently, de Jong et al. have revealed a notable diversification of the IFN signature among patients with immune-mediated diseases with regards to the ratio between IFNα- and IFNβ-specific response programs (18). Interestingly, RA patients exhibited an intermediate position between SLE patients (mostly IFNα specific) and IFNβ-treated multiple sclerosis patients (mostly IFNβ specific). Importantly, MX1 was identified as one of the IFNβ-related genes. However, the clinical relevance of this IFNα/β ratio was not analyzed. Overall, all these findings reinforce the idea that not only the degree of activation of the type I IFN but also its composition are key to unveil its potential role as biomarker in RA.

In conclusion, our results revealed that the IFN signature was not quantitatively and qualitatively homogeneous in RA, but certain heterogeneity can be recognized. Both the disease course and therapies are associated with changes in the levels of expression and the structure of the IRGs response in RA, hence limiting its clinical relevance. To the best of our knowledge, this is the first study where the peripheral blood type I IFN signature, together with its clinical relevance, was quantitatively and qualitatively analyzed in VERA. Although this work represents a proof-of-concept study of the type I IFN signature in RA and its clinical relevance, it paves the ground for future, larger studies involving a higher number of IRG and long-term clinical outcomes. This will allow the identification of the best panel of IRGs and the best approach to be implemented in the context of personalized medicine.

## Ethics Statement

The study was approved by the Institutional Review Board (Comité de Ética de Investigación Clínica del Principado de Asturias) in compliance with the Declaration of Helsinki. All study subjects gave written informed consent.

## Author Contributions

JR-C performed most of the experimental procedures, carried out the statistical analyses and drafted and edited the manuscript. PL performed some experimental procedures. MA-L and FB-G were in charge of patients’ recruitment and clinical data collection and management. AS conceived the study, designed the protocols and drafted and edited the manuscript. All authors read and approved the final version of the manuscript.

## Conflict of Interest Statement

The authors declare no conflicts of interest. Funding bodies had no role in study conception, design, experimental procedures, analysis, or decision to publish.

## Abbreviations

ACPA, anticitrullinated peptide antibodies; bDMARDs, biological DMARDs; CRP, C-reactive protein; csDMARDs, conventional synthetic DMARDs; DAS28, disease activity score 28-joints; DMARDs, disease-modifying antirheumatic drugs; EULAR, European league against rheumatism; ESR, erythrocyte sedimentation rate; HAQ, health assessment questionnaire; HC, healthy control; IFI6, interferon alpha inducible protein 6; IFI44, interferon-induced protein 44; IFI44L, interferon-induced protein 44 like; IFN, interferon; IRG, interferon-responding gene; MHC, major histocompatibility factor; MX1, MX dynamin-like GTPase 1; NSAIDs, non-steroidal anti-inflammatory drugs; RA, rheumatoid arthritis; RF, rheumatoid factor; TNF, tumor necrosis factor alpha.
