# Supplementary figures and images for: Heterogeneity of the Type I Interferon Signature in Rheumatoid Arthritis: A Potential Limitation for Its Use As a Clinical Biomarker
Source: Front Immunol. 2018 Jan 16;8:2007. doi: 10.3389/fimmu.2017.02007 (PMC5775969; doi:10.3389/fimmu.2017.02007)

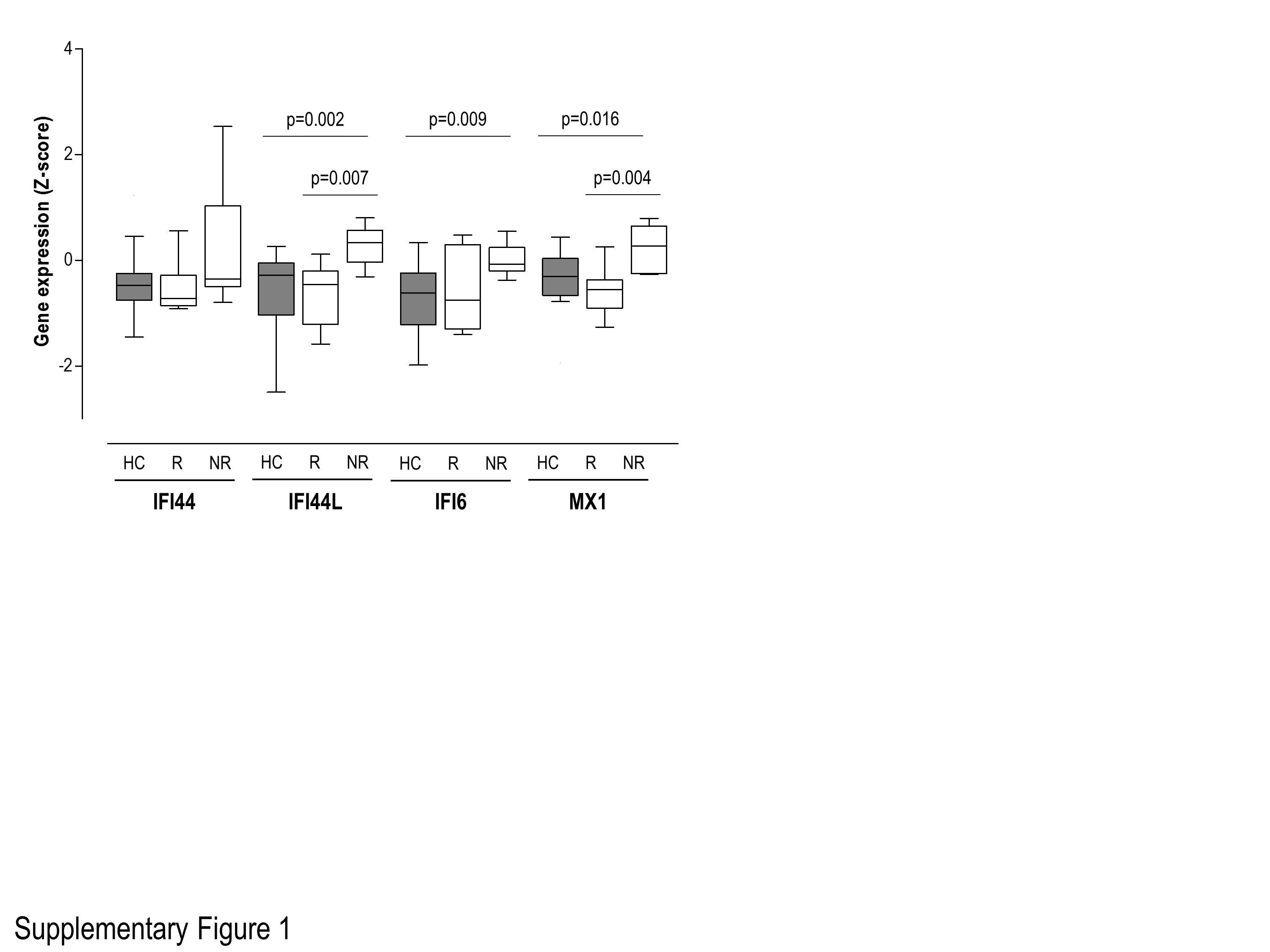

Supplement: Supplementary file 1 [file Image_1.tif]
