# Supplementary material for: Heterogeneity of the Type I Interferon Signature in Rheumatoid Arthritis: A Potential Limitation for Its Use As a Clinical Biomarker
Source: Front Immunol. 2018 Jan 16;8:2007. doi: 10.3389/fimmu.2017.02007 (PMC5775969; doi:10.3389/fimmu.2017.02007)
Supplement: Supplementary file 2 [file Table_1.docx]

**SUPPLEMENTARY MATERIAL**

**Supplementary Table 1: Clinical and demographical characteristics of the RA patients recruited in this study stratified by their disease status.**

|  | VERA  (n=18) | bDMARD-naïve  (n=44) | bDMARD  (n=36) | p-value |
| --- | --- | --- | --- | --- |
| **Demographical features** |  |  |  |  |
| Age, years; median (range) | 52.08 (31.50 – 64.50) | 52.79 (22.42 – 65.10) | 57.41 (22.00 – 65.00) | 0.439 |
| Gender, f/m | 13/5 | 37/7 | 29/7 | 0.778 |
| **Disease features** |  |  |  |  |
| Disease duration, years | - | 3.29 (4.90) | 8.16 (8.67) | - |
| Age at diagnosis, years; median (range) | 52.08 (31.50 – 63.00) | 48.25 (19.00 – 65.00) | 45.33 (20.00 – 62.00) | 0.473 |
| Disease activity (DAS28) | 5.10 (1.55) | 3.46 (2.43) | 3.70 (1.42) | <0.001 |
| Tender Joint Count | 8.00 (4.00) | 2.00 (6.25) | 2.00 (3.00) | <0.001 |
| Swollen Joint Count | 5.00 (2.00) | 1.00 (4.00) | 1.00 (2.00) | <0.001 |
| Patient Global Assessment (0-100) | 65.00 (24.00) | 38.00 (49.25) | 40.00 (30.00) | 0.031 |
| ESR, mm/h | 26.00 (33.00) | 12.00 (20.00) | 19.00 (21.00) | 0.268 |
| CRP, mg/l | 3.50 (9.25) | 2.00 (2.70) | 2.00 (4.00) | 0.549 |
| HAQ (0-3) | 1.37 (1.21) | 0.50 (1.26) | 1.00 (0.82) | 0.117 |
| RF (+), n(%) | 11 (61.1) | 24 (54.4) | 23 (63.8) | 0.608 |
| ACPA (+), n(%) | 11 (61.1) | 25 (56.8) | 25 (69.4) | 0.183 |
| Erosive disease, n(%) | 1 (5.5) | 16 (36.3) | 24 (66.6) | <0.001 |
| Treatments, n(%) |  |  |  |  |
| None | 18 (100) | 0 (0) | 0 (0) | - |
| Glucocorticoids | 0 (0) | 29 (65.9) | 27 (75.0) | - |
| Methotrexate | 0 (0) | 39 (88.6) | 26 (72.2) | - |
| TNFα blockers | 0 (0) | 0 (0) | 36 (100) | - |

Variables were summarized as median (interquartile range) or n(%), as appropriate, unless otherwise stated. Differences in demographic parameters were assessed by Kruskal-Wallis tests or χ2 tests, according to the distribution of the variables.

**Supplementary Table 2: Leukocyte populations in peripheral blood of the subjects recruited in this study.** Total counts of leukocytes, neutrophils, lymphocytes and monocytes for HC and RA patients depending on their clinical stage are shown.

|  | **HC** | **VERA** | **bDMARD-naïve** | **bDMARD** |
| --- | --- | --- | --- | --- |
| Leukocytes (·10^3^/μl) | 6.52±1.37 | 8.02±2.07 | 7.17±2.29 | 7.76±2.27 |
| Neutrophils (·10^3^/μl) | 3.73±1.50 | 5.15±1.95 | 4.76±2.32 | 5.05±2.4 |
| Lymphocytes (·10^3^/μl) | 1.99±0.42 | 1.90±0.83 | 1.60±0.54 | 1.78±0.64 |
| Monocytes (·10^3^/μl) | 0.45±0.12 | 0.53±0.18 | 0.46±0.17 | 0.60±0.86 |

**Supplementary Table 3: IRG expression upon TNFα-blockade in RA patients prospectively followed up for 3 months.**

|  |  | **BL** | **PT** | **p-value** |
| --- | --- | --- | --- | --- |
| **Responders** | IFI44 | -0.51 (0.49) | -0.42 (1.37) | 0.500 |
|  | IFI44L | -0.14 (0.38) | -0.31 (1.23) | 0.893 |
|  | IFI6 | -0.14 (0.66) | -0.23 (1.68) | 0.345 |
|  | MX1 | -0.20 (1.08) | -0.24 (1.79) | 0.686 |
| **Moderate/non-responders** | IFI44 | -0.56 (1.79) | -0.25 (1.99) | 0.889 |
|  | IFI44L | -0.19 (1.40) | -0.45 (2.00) | 0.575 |
|  | IFI6 | -0.22 (1.52) | 0.44 (1.76) | 0.575 |
|  | MX1 | -0.13 (1.22) | -0.31 (2.06) | 0.779 |

**Supplementary Figure 1: Expression of IRGs and clinical outcome in VERA patients.** Analysis of the individual IRGs expression in VERA patients (white boxes) compared to HC (gray boxes). and RA patients classified as responders (R) or non-responders (NR) according to EULAR criteria after 6 months. Boxes represent 25^th^ and 75^th^ percentiles, whereas whiskers represent minimum and maximum values. Statistical analyses were performed by Kruskal-Wallis with Dunn-Bonferroni tests for multiple comparisons. P-values correspond to those obtained in the multiple comparisons tests.
